# The Wako Cohort Study: Design and Profile of Participants at Baseline

**DOI:** 10.2188/jea.JE20240288

**Published:** 2025-07-05

**Authors:** Yuri Yokoyama, Yu Nofuji, Takumi Abe, Kumiko Nonaka, Yumi Ozone, Yuka Nakamura, Shiina Chiaki, Takumi Suda, Naoko Saito, Mai Takase, Hidenori Amano, Susumu Ogawa, Hiroyuki Suzuki, Hiroshi Murayama

**Affiliations:** Research Team for Social Participation and Healthy Aging, Tokyo Metropolitan Institute for Geriatrics and Gerontology, Tokyo, Japan

**Keywords:** cohort profile, healthy aging, health disparities, healthy life expectancy

## Abstract

**Background:**

We launched the Wako Cohort Study in 2023 to identify individual and socio-environmental factors related to the extension of healthy life expectancy and the reduction of health disparities among community-dwelling adults and to develop health promotion and care prevention strategies. This study profile aims to describe the study design and participants’ profile at baseline.

**Methods:**

The Wako Cohort Study is a prospective study of community-dwelling adults aged ≥40 years living in Wako City, Saitama Prefecture, Japan. The Wako Cohort Study consists of two surveys: a mail-in survey for persons aged ≥40 years and a face-to-face assessment (on-site survey) for those aged ≥65 years. The survey items were designed considering the following points: 1) life course perspective (transition from middle to old age in the life course), 2) health indifference, and 3) employment in older age.

**Results:**

A total of 8,824 individuals participated in the mail-in survey (2,395 persons aged 40–64 years and 6,429 aged ≥65 years). Of those aged ≥65 years who returned the mail survey, 1,004 participated in the subsequent on-site survey. Men aged ≥65 years tended to have higher health interests than those aged 40–64 years; however, this was not true for women. In the mail-in survey, 30.4% of those aged ≥65 years were employed.

**Conclusion:**

The Wako Cohort Study is expected to provide new insights into the development of strategies to extend healthy life expectancy and reduce health disparities in Japan.

## PURPOSE

With the increase in global population aging, extending healthy life expectancy and reducing health disparities have become important public health issues. In Japan, the National Health Promotion Program, known as Health Japan 21 (the third term), was launched in 2024. It set four basic goals for the period 2024–2035: 1) to extend healthy life expectancy and reduce health disparities, 2) to improve individual behavior and health status, 3) to improve the quality of the social environment, and 4) to promote health based on a life-course approach.^1^

Numerous cohort studies targeting community-dwelling older people have been launched in Japan to identify risk factors for lifestyle-related diseases and to elucidate factors that extend healthy life expectancy. We have also conducted several prospective cohort studies that focused on the prevention of frailty and functional disability among community-dwelling older Japanese people.^2^^–^^6^ However, challenges remain because these cohort studies failed to consider the following three points. First, a life-course perspective is crucial for promoting healthy aging.^7^^,^^8^ Therefore, in this cohort study, we decided to focus on not only the older population but also the middle-aged population. This is because one of the results of our cohort study among older adults, which examined the trajectories of various functions (eg, higher-level functional capacity and the frailty score) with aging, revealed that differences were already present at the age of 65 years.^9^^,^^10^ Additionally, in the British birth cohort study, lower midlife physical capacity was associated with a higher mortality rate.^11^ These results suggest the importance of seamless observation of health conditions from midlife to later life and the need to start preventing frailty and long-term care before old age. Second, to reduce health disparities, it is not sufficient to target those who are interested in health promotion and frailty prevention and can actively participate in these activities; thus, understanding the characteristics of populations with health indifference will help to develop effective approaches to promote changes in awareness and behavior among those who are indifferent to health promotion and social participation. Finally, there is growing interest in the employment of older adults as a countermeasure to the labor shortage resulting from the declining birth rate and aging population. Therefore, research on the promotion of diverse employment among older adults would be helpful in building a sustainable society.

Given this background, we launched the Wako Cohort Study in 2023. This study aimed to include middle-aged and older adults to identify individual and socio-environmental factors related to the extension of healthy life expectancy and reduction of health disparities among community-dwelling adults, as well as effective population-based health promotion and preventive long-term care strategies that can be applied to diverse populations. Wako City and the Tokyo Metropolitan Institute for Geriatrics and Gerontology have collaborated to facilitate health promotion and care prevention under a memorandum of understanding since 2022. Wako City tends to have a higher percentage of people in the age group 20–50 years than the rest of Japan, making health promotion and care prevention from young and middle age and measures for populations with health indifference a major issue.^12^ Moreover, Wako City has positioned the promotion of social participation, including employment, of older adults as a major policy for care prevention.^13^ To strengthen these efforts and generate evidence that can be applied in other municipalities, three points were prioritized for this cohort: 1) life-course perspective (transition from middle to old age in the life course), 2) health indifference, and 3) employment in older age. Here, this paper describes the study design and profile of the participants at baseline.

## MAIN FEATURES

The Wako Cohort Study is a prospective study of community-dwelling adults aged ≥40 years living in Wako City, Saitama Prefecture, Japan. The main features of the Wako Cohort Study include the following three considerations in the study design and survey items: 1) life-course perspective (transition from middle to old age in the life course), 2) health indifference, and 3) employment in older age. First, to focus on the transition from middle to old age in the life course, the Wako Cohort Study consists of a mail-in survey for those aged ≥40 years and an on-site survey for those aged ≥65 years. Second, survey items were added to identify the level of health indifference. Finally, in addition to adding a variety of items related to employment in older age to the questionnaire survey, a face-to-face interview (on-site survey) was conducted with those aged ≥65 years to allow for a detailed assessment of items related to employment. Although this cohort study is primarily based on a mail-in survey, on-site surveys for some items were incorporated for participants aged ≥65 years to reduce bias in health outcomes and employment status, which are difficult to assess using only questionnaire surveys.

### Participants

**Figure 1.**
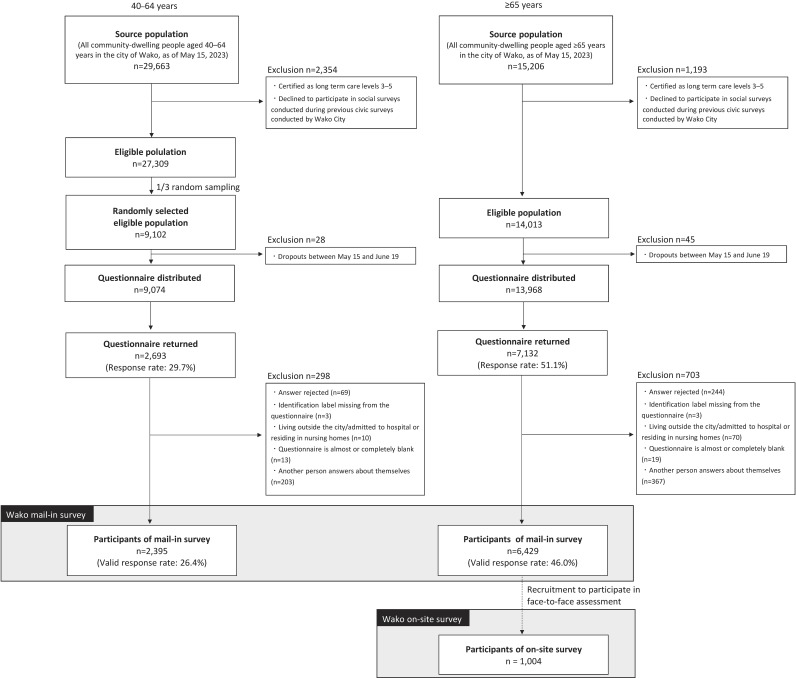
Flow diagram of study participants: the Wako Cohort Study, 2023

In this prospective study, the source population comprised community-dwelling individuals aged ≥40 years who were included in the Residential Registry for Wako City, Saitama Prefecture, Japan. Wako City is located in the southern part of Saitama Prefecture and is a suburban area located 19 km from central Tokyo. As of May 1, 2023, it had a population of 84,267 (43,239 men and 41,028 women), a population density of 7,633 persons/km^2^, and an aging rate of 18.0%. Figure 1 shows the flow diagram of the study participants. The baseline mail-in survey was conducted from June to August 2023 using a self-administered questionnaire, and the on-site survey was conducted from September to October 2023 using a face-to-face assessment. The on-site survey was conducted at six community centers in Wako City. Financial incentives were offered to encourage participation in the on-site survey, and participants received feedback on their individual results afterward.

The mail-in survey was conducted in two age groups: persons aged 40–64 years (*n* = 29,663 as of May 15, 2023) and those aged ≥65 years (*n* = 15,206 as of May 15, 2023). In the mail-in survey, we excluded people with long-term care insurance (LTCI) certification levels 3–5 and those who had declined to participate in social surveys during previous civic surveys conducted by Wako City. For persons aged 40–64 years, we randomly selected one-third of the eligible population (*n* = 27,309); thus, 9,102 were selected. For persons aged ≥65 years, 14,013 were eligible for inclusion. After excluding those who died or were transferred between May 15 and the date of the questionnaire dispatched in June, 9,074 and 13,968 questionnaires were distributed. Additionally, for those aged ≥65 years, an invitation to participate in a face-to-face assessment was enclosed with a mailed survey to recruit participants for the on-site survey.

The questionnaire included an identification number so that the data from the mail-in and on-site surveys could be combined with the data from the follow-up survey (described below). To reduce response bias in the mail-in survey, a reminder was sent in July 2023 to all non-respondents.

The study protocol was approved by the Ethics Committee of the Tokyo Metropolitan Institute for Geriatrics and Gerontology (approved on May 30, 2023; approval number R23-006). All participants provided informed consent before their inclusion in the study. In the mail-in survey, a statement detailing the study objectives, voluntary participation, and guarantee of anonymity in the analysis were appended to the questionnaire. Written informed consent was obtained from all participants of the on-site survey.

### Outcomes and follow-up

We plan to include three outcomes: survival time, LTCI certification, and long-term care costs. To monitor these outcomes from the baseline survey, we will confirm information on the date of death or movement out of the study area, LTCI certification status (date and level of LTCI certification), and monthly long-term care costs. This information will be provided by Wako City. Furthermore, to track changes in each participant’s health and lifestyle, we will perform a follow-up survey in the same way as the baseline survey every few years. We set the initial study period to 5 years and intend to extend it as necessary.

### Measurements

Table 1 shows the main measures surveyed at baseline. The baseline questionnaire of the mail-in survey for persons aged 40–64 years was the same as that for those aged ≥65 years, excluding the Kihon Checklist.^14^

**Table 1.** Summary of items on the Wako Cohort Study at baseline, 2023

	Mail-in survey		On-site survey
	40–64 years	≥65 years	≥65 years
**Demographics**			
Living arrangement	○	○	^*^
Socioeconomic status	○	○	^*^
**Medical and lifestyle characteristics**			
Medical history	○	○	^*^
Smoking, alcohol consumption, physical activity, and dietary habits	○	○	^*^
Body mass index	○ (Self reported)	○ (Self reported)	○ (Actual measured)
**Mental health/psychological characteristics**			
Self-rated health	○	○	^*^
Depression (two-question case-finding instrument)	○	○	^*^
Depressive mood (5-item Geriatric Depression Scale; GDS-5)			○
Loneliness (3-item UCLA loneliness scale)	○	○	^*^
Well-being (WHO-Five Well-being Index)	○	○	^*^
Health indifference (Health Interest Scale)	○	○	^*^
Procrastination (General Procrastination Scale)			○
Personality traits (Japanese version of the Ten-Item Personality Inventory)			○
**Frailty**			
Frailty (Kihon checklist)		○	^*^
**Social factors**			
Frequency of outings		○	^*^
Social networks (social isolation), social support	○	○	^*^
Social participation	○	○	^*^
Paid work (current job, jobs engaged in for the longest period of time, and intention to work)	○	○	○
**Environmental factors**			
Social environment (social capital)	○	○	^*^
Physical activity environment	○	○	
Food environment	○	○	○
**Body composition**			
Fat mass, fat-free mass, and skeletal muscle mass			○
**Physical function**			
Grip strength, usual walking speed, balance tests, and Five Times Sit to Stand Test			○
**Cognitive function**			
Cognitive function (Japanese version of the Montreal Cognitive Assessment; MoCA-J)			○

UCLA, University of California, Los Angeles; WHO, World Health Organization.

^a^As the participants in the on-site survey are also participants in the mail-in survey, items collected in the mail-in survey are used in the analysis.

#### Demographics

We assessed living arrangements and socioeconomic status. Socioeconomic status included educational attainment and annual household income. Information on the districts (chocho-aza level) where the participants resided was obtained from Wako City.

#### Medical and lifestyle characteristics

We assessed medical history (hypertension, heart disease, cerebrovascular diseases, cancer, diabetes mellitus, liver disease, periodontal disease, and chronic lower back and nerve pain), smoking status, alcohol consumption, physical activity, food intake frequency, and self-reported height and weight. Occupational, transport-related, and leisure-time physical activity were assessed using questions based on the Global Physical Activity Questionnaire (GPAQ).^15^ We adapted the original question about occupational physical activity in the GPAQ by dividing it into work-related and household physical activity, referring to the International Physical Activity Questionnaire.^16^ Moreover, the food intake frequencies of 15 types of food groups were inquired. The dietary variety score was calculated using the intake frequency of 10 food groups (meat, fish/shellfish, eggs, milk, soybean products, green/yellow vegetables, potatoes, fruit, seaweed, and fats/oils).^17^ In the mail-in survey, the body mass index (BMI) was calculated as the self-reported body weight (in kg) divided by the self-reported body height (in m) squared (kg/m^2^). In the on-site survey, the BMI was calculated using the aforementioned formula based on actual height and weight measurements.

#### Mental health/psychological characteristics

This included self-rated health, depression (the two-question case-finding instrument for depression),^18^ depressive mood (the 5-item Geriatric Depression Scale),^19^^,^^20^ loneliness (the University of California, Los Angeles [UCLA] Loneliness Scale, version 3, Short Form 3-item scale),^21^ well-being (the World Health Organization-Five Well-being Index),^22^^,^^23^ health indifference (the Health Interest Scale [HIS]),^24^ procrastination (the Japanese version of the General Procrastination Scale),^25^ and personality (the Japanese version of the Ten-Item Personality Inventory).^26^

#### Frailty

Frailty was assessed using the Kihon Checklist (range 0–25),^14^ with a cutoff of ≥8 points.^27^^,^^28^

#### Social factors

We assessed the frequency of outings, social networks (social isolation), social support, social participation, and paid work. As a measure of social networks, we inquired about the frequency of contact with family, relatives, and friends, apart from cohabitating family members and business associates. Social support inquired about the availability of instrumental and emotional support. We asked about participation in volunteer groups, sports groups or clubs, hobby groups, learning or cultural groups, groups for preventing long-term care (in persons aged ≥65 years), senior citizens’ clubs (in persons aged ≥65 years), neighborhood associations, and other groups. In addition, paid work included current job conditions (frequency, working time, and employment status), jobs engaged in for the longest period of time, and the intention to work (eg, job preferences, what is important to you when choosing a job). To expand employment opportunities for older adults, the employment needs (desired type and content of employment) were asked about, regardless of whether or not the person was currently employed.

#### Environmental factors

This included the social, physical-activity, and food environments. The social environment included social capital (neighborhood trust and neighborhood norms). Physical-activity environment factors were assessed by asking respondents about their perception of their physical activity environment using several items, including accessibility by foot/bicycle/public transport. Food environment factors were assessed using self-reported questionnaires, including the frequency of food purchases and the stores that are frequently used to purchase groceries. These items of physical activity and food environment factors were developed for this study.

#### Body composition

We assessed fat, fat-free, and skeletal muscle mass. Body composition parameters were measured using a direct segmental multifrequency bioelectrical impedance analysis (InBody 770 analyzer; InBody Co. Ltd, Seoul, Korea), which uses a tetrapolar, eight-point tactile electrode system that separately measures the impedance of the arms, trunk, and legs at six different frequencies (1, 5, 50, 250, 500, and 1,000 kHz).

#### Physical function

This included grip strength, usual walking speed, balance tests, and the 5-time chair stand test. Grip strength was measured twice for each hand using a Smedley-type hand dynamometer. Gait speed was evaluated as the time (in seconds) taken for a 4-, 5-, and 6-m walk. For the balance test, participants were asked to stand in side-by-side semi-tandem and tandem stances for 10 seconds. For the five-time chair stand test, participants were instructed to perform sit-to-stand movements five times as quickly as they could, keeping their arms crossed over their chests. These measurements were conducted by well-trained staff.

#### Cognitive function

This was assessed using the Japanese version of the Montreal Cognitive Assessment (MoCA-J).^29^ The possible scores ranged from 0 to 30, with higher scores indicating more favorable cognitive performance. We defined mild cognitive impairment as a MoCA-J score of ≤25 points.^29^ Cognitive function was assessed by well-trained staff.

## BASELINE CHARACTERISTICS

In total, 2,693 persons aged 40–64 years returned the questionnaires (response rate: 29.7%), and 2,395 were valid responses (valid response rate: 26.4%). In the survey of people aged ≥65 years, 7,132 returned the questionnaires (response rate: 51.1%), and 6,429 were valid responses (valid response rate: 46.0%). Of those who returned the mail survey, 1,004 participated in the on-site survey (Figure 1).

Table 2 presents the baseline characteristics of the participants in the mail-in survey. The mean age of participants was 52.6 (standard deviation [SD], 7.0) years for the 40–64-year age group and 76.5 (SD, 6.8) years for the ≥65-year age group. The proportion of men was 43.9% in the 40–64-year age group and 45.0% in the ≥65-year age group. Being widowed or divorced and living alone was more prevalent in women aged ≥75 years than in the other groups. In people aged ≥65 years, an education duration of ≥13 years was more prevalent in men than in women, but there was almost no sex difference in those aged 40–64 years.

**Table 2.** Baseline characteristics of the participants by age and sex: the Wako mail-in survey, 2023

		All	Men			Women		
	
			40–64 years	65–74 years	≥75 years	40–64 years	65–74 years	≥75 years
Number of individuals		8,824	1,052	1,270	1,621	1,343	1,466	2,072
**Demographics**								
Age	Mean (SD)	70.0 (12.7)	52.8 (7.1)	70.2 (2.7)	80.9 (4.9)	52.5 (7.0)	70.3 (2.7)	81.4 (5.0)
Sex	Men, *n* (%)	3943 (44.7)	—	—	—	—	—	—
Living alone	Yes, *n* (%)	1,634 (18.8)	172 (16.5)	235 (18.8)	237 (14.9)	140 (10.6)	239 (16.5)	611 (30.3)
Marital status	Married, *n* (%)	6,095 (69.8)	791 (75.3)	991 (78.9)	1,283 (80.6)	1,050 (78.2)	1,052 (72.4)	928 (45.6)
Years of education	≥13, *n* (%)	4,695 (54.3)	765 (72.8)	804 (64.1)	788 (50.1)	959 (71.8)	769 (53.1)	610 (30.8)
Equivalent income	≥2.0 million yen, *n* (%)	4,671 (52.9)	872 (82.9)	789 (62.1)	732 (45.2)	956 (71.2)	668 (45.6)	654 (31.6)
**Medical and lifestyle characteristics**								
Number of chronic diseases	0, *n* (%)	2,250 (27.8)	462 (51.3)	273 (22.9)	203 (13.3)	690 (57.3)	347 (26.2)	275 (14.2)
	1, *n* (%)	3,430 (42.5)	309 (34.3)	526 (44.1)	622 (40.8)	401 (33.3)	627 (47.4)	945 (48.9)
	≥2, *n* (%)	2,400 (29.7)	130 (14.4)	393 (33.0)	699 (45.9)	114 (9.5)	350 (26.4)	714 (36.9)
Smoking	Current smoker, *n* (%)	864 (10.0)	240 (23.0)	246 (19.6)	148 (9.3)	104 (7.8)	78 (5.4)	48 (2.4)
Alcohol consumption	Current alcohol consumption, *n* (%)	4,348 (50.0)	755 (72.5)	872 (69.3)	945 (59.1)	698 (52.6)	599 (41.3)	479 (23.7)
Engaging in any exercise more than once a week	Yes, *n* (%)	5,398 (62.4)	647 (61.7)	822 (66.5)	1,032 (65.2)	675 (50.3)	954 (66.3)	1,268 (63.3)
Dietary variety score (range: 0–10)	Mean (SD)	3.4 (2.7)	2.0 (2.1)	2.6 (2.4)	3.5 (2.7)	3.0 (2.3)	3.8 (2.6)	4.6 (2.8)
Body mass index, kg/m^2^	Mean (SD)	22.7 (3.5)	24.0 (3.5)	23.5 (3.0)	23.0 (3.0)	22.1 (3.9)	22.2 (3.4)	22.2 (3.7)
**Mental health/psychological characteristics**								
Self-rated health	Good, *n* (%)	7,037 (81.8)	849 (81.9)	1,023 (82.2)	1,226 (77.7)	1,125 (85.2)	1,242 (87.1)	1,572 (78.5)
Depression	Presence, *n* (%)	2,492 (29.2)	349 (33.6)	278 (22.4)	442 (28.4)	444 (33.5)	345 (24.1)	634 (32.6)
3-item UCLA loneliness scale (range: 3–12)	Mean (SD)	5.9 (2.0)	6.6 (2.1)	6.0 (1.9)	5.9 (2.0)	6.4 (2.0)	5.6 (1.9)	5.5 (1.9)
WHO-Five Well-being Index (range: 0–25)	Mean (SD)	14.9 (5.6)	14.2 (5.5)	15.3 (5.4)	15.1 (5.9)	14.5 (5.2)	15.4 (5.4)	14.8 (5.8)
Health Interest Scale (range: 12–48)	Mean (SD)	36.1 (5.4)	33.9 (5.9)	36.0 (5.6)	36.7 (5.4)	35.5 (5.1)	36.8 (4.8)	36.8 (5.2)
**Frailty**								
Kihon checklist (range: 0–25)	Score ≥8 (frailty), *n* (%)	1,261 (24.3)	—	209 (18.4)	397 (31.0)	—	189 (14.4)	466 (31.9)
**Social factors**								
Outing ≥5 days a week	Yes, *n* (%)	2,857 (44.7)	—	745 (58.9)	670 (41.6)	—	771 (52.8)	671 (32.6)
Social isolation	Presence, *n* (%)	2,413 (30.2)	112 (10.9)	434 (35.7)	760 (53.3)	132 (10.0)	336 (25.1)	639 (38.5)
Social support	Receiving instrumental support, *n* (%)	7,759 (89.6)	889 (85.2)	1,084 (87.1)	1,450 (91.0)	1,209 (91.4)	1,347 (92.8)	1,780 (88.7)
	Receiving emotional support, *n* (%)	8,126 (93.6)	908 (87.1)	1,118 (89.7)	1,445 (91.1)	1,279 (96.5)	1,429 (97.9)	1,947 (96.2)
Social participation, more than once a month	Yes, *n* (%)	3,273 (38.2)	220 (21.1)	430 (34.7)	589 (38.0)	362 (27.1)	689 (47.9)	983 (50.1)
Current job	Regular employee, *n* (%)	1,404 (16.7)	747 (73.1)	144 (11.7)	50 (3.3)	393 (29.9)	50 (3.5)	20 (1.1)
	Non-regular employee, *n* (%)	1,765 (21.0)	94 (9.2)	379 (30.8)	170 (11.2)	593 (45.1)	399 (28.2)	130 (6.9)
	Self-employee, *n* (%)	468 (5.6)	90 (8.8)	130 (10.6)	113 (7.5)	44 (3.3)	49 (3.5)	42 (2.2)
	Family-employee, *n* (%)	75 (0.9)	2 (0.2)	3 (0.2)	6 (0.4)	14 (1.1)	26 (1.8)	24 (1.3)
	Other, *n* (%)	135 (1.6)	17 (1.7)	32 (2.6)	20 (1.3)	14 (1.1)	24 (1.7)	28 (1.5)
	Unemployed, *n* (%)	4,541 (54.1)	72 (7.0)	542 (44.1)	1156 (76.3)	257 (19.5)	865 (61.2)	1,649 (87.1)
Intention to work	Yes, *n* (%)	2,600 (33.8)	586 (58.4)	442 (38.3)	219 (16.2)	804 (63.3)	403 (30.2)	146 (9.3)
**Environmental factors**								
Neighborhood trust	Agree or somewhat agree, *n* (%)	4,059 (47.7)	371 (35.6)	555 (44.9)	832 (54.4)	575 (43.1)	676 (47.3)	1,050 (54.4)
Neighborhood norms	Agree or somewhat agree, *n* (%)	3,158 (37.7)	279 (26.7)	433 (35.3)	629 (41.7)	461 (34.6)	531 (37.7)	825 (44.3)

SD, standard deviation; UCLA, University of California, Los Angeles; WHO, World Health Organization.

Missing values are removed.

Current smoking and alcohol consumption were more prevalent in men than in women in both age groups. The dietary variety score was higher among women in both age groups than in men. The proportion of those engaging in any exercise more than once a week was higher in the ≥65-year age group than in the 40–64-year age group for both men and women, and it was lowest among women aged 40–64 years. Depression was more prevalent in the 40–64-year age group for men and in the 40–64- and ≥75-year age groups for women than in the other age groups. The HIS scores tended to be higher for men aged ≥65 years than for men aged 40–64 years but remained comparable for women across age groups. Frailty was more prevalent in people aged ≥75 years than in those aged 65–74 years in both men and women.

No apparent differences in loneliness were observed according to age group or sex; however, social isolation was more prevalent among men aged ≥75 years than in the other groups. Social participation was more frequent in the ≥65-year age group than in the 40–64-year age group in both men and women. The proportion of those who were employed was lower in the older age groups than in the younger age groups. Regarding current employment status, the number of regular employees was higher among men aged 40–64 years, whereas the number of non-regular employees was higher among women aged 40–64 years and both sexes aged ≥65 years. The proportion of people answering “agree or somewhat agree” to neighborhood trust and norms was higher in the group aged ≥75 years than in those aged 40–64 and 65–74 years for both men and women.

Table 3 shows the baseline characteristics of the participants in the on-site survey. The mean age of the participants in the on-site survey was 75.3 (SD, 6.4) years, and 48.1% of participants were men. Demographic variables, such as sex, age, and living arrangements, did not substantially differ between the mail-in and on-site surveys; the medical and lifestyle characteristics in the on-site survey were more favorable than those in the mail-in survey. In addition, social isolation was lower in the on-site survey than in the mail-in survey. Similarly, social participation was more frequent in the on-site survey than in the mail-in survey.

**Table 3.** Baseline characteristics of the participants by age and sex: the Wako on-site survey, 2023

		All	Men		Women	
	
			65–74 years	≥75 years	65–74 years	≥75 years
Number of individuals		1,004	236	247	260	261
**Demographics**						
Age	Mean (SD)	75.3 (6.4)	70.1 (2.7)	80.2 (4.5)	70.0 (2.9)	80.8 (4.4)
Sex	Men, *n* (%)	483 (48.1)	—	—	—	—
Living alone	Yes, *n* (%)	219 (21.8)	37 (15.7)	34 (13.8)	47 (18.1)	101 (38.8)
Marital status	Married, *n* (%)	703 (70.0)	195 (82.6)	202 (81.8)	194 (74.6)	112 (42.9)
Years of education	≥13, *n* (%)	612 (61.0)	185 (78.4)	162 (65.6)	160 (61.5)	105 (40.2)
Equivalent income	≥2.0 million yen, *n* (%)	554 (55.2)	168 (71.2)	142 (57.5)	137 (52.7)	107 (41.0)
**Medical and lifestyle characteristics**						
Number of chronic diseases	0, *n* (%)	244 (24.4)	64 (27.4)	46 (18.6)	77 (29.7)	57 (21.8)
	1, *n* (%)	429 (42.9)	92 (39.3)	104 (42.1)	121 (46.7)	112 (42.9)
	≥2, *n* (%)	328 (32.8)	78 (33.3)	97 (39.3)	61 (23.6)	92 (35.2)
Smoking	Current smoker, *n* (%)	66 (6.6)	35 (14.8)	21 (8.5)	6 (2.3)	4 (1.5)
Alcohol consumption	Current alcohol consumption, *n* (%)	533 (53.1)	166 (70.3)	161 (65.2)	125 (48.1)	81 (31.0)
Engaging in any exercise more than once a week	Yes, *n* (%)	776 (77.4)	178 (75.4)	189 (76.5)	188 (72.3)	221 (85.0)
Dietary variety score (range: 0–10)	Mean (SD)	3.8 (2.7)	2.7 (2.3)	3.7 (2.7)	4.0 (2.6)	4.8 (2.8)
Body mass index, kg/m^2^	Mean (SD)	22.4 (3.0)	23.4 (3.0)	22.7 (2.6)	21.8 (3.1)	21.8 (3.1)
**Mental health/psychological characteristics**						
Self-rated health	Good, *n* (%)	863 (86.1)	203 (86.0)	207 (84.1)	228 (87.7)	225 (86.5)
Depression	Presence, *n* (%)	244 (24.3)	43 (18.2)	50 (20.3)	74 (28.5)	77 (29.6)
GDS-5	Score ≥2 (depressive mood), *n* (%)	275 (27.4)	57 (24.2)	61 (24.9)	83 (31.9)	74 (28.5)
3-item UCLA loneliness scale (range: 3–12)	Mean (SD)	5.7 (1.9)	6.0 (1.9)	5.6 (2.0)	5.6 (1.9)	5.6 (2.0)
WHO-Five Well-being Index (range: 0–25)	Mean (SD)	15.7 (5.2)	15.7 (4.8)	15.7 (5.5)	15.6 (4.9)	16.0 (5.4)
Health Interest Scale (range: 12–48)	Mean (SD)	38.2 (4.8)	37.9 (5.2)	38.9 (4.8)	37.8 (4.4)	38.3 (4.8)
**Frailty**						
Kihon checklist (range: 0–25)	Score ≥8 (frailty), *n* (%)	181 (18.7)	38 (16.3)	47 (19.9)	29 (11.6)	67 (27.0)
**Social factors**						
Outing ≥5 days a week	Yes, *n* (%)	543 (54.1)	144 (61.0)	139 (56.3)	149 (57.3)	111 (42.5)
Social isolation	Presence, *n* (%)	318 (31.8)	77 (32.6)	113 (45.7)	49 (18.8)	79 (30.6)
Social support	Receiving instrumental support, *n* (%)	868 (89.4)	206 (88.4)	221 (92.1)	230 (92.4)	211 (84.7)
	Receiving emotional support, *n* (%)	913 (93.9)	211 (90.6)	220 (91.7)	244 (97.6)	238 (95.6)
Social participation, more than once a month	Yes, *n* (%)	635 (63.2)	120 (50.8)	148 (59.9)	168 (64.6)	199 (76.2)
Current job	Regular employee, *n* (%)	36 (3.6)	19 (8.1)	10 (4.0)	5 (1.9)	2 (0.8)
	Non-regular employee, *n* (%)	222 (22.1)	76 (32.2)	33 (13.4)	87 (33.5)	26 (10.0)
	Self-employee, *n* (%)	54 (5.4)	23 (9.7)	15 (6.1)	8 (3.1)	8 (3.1)
	Family-employee, *n* (%)	9 (0.9)	1 (0.4)	0 (0.0)	6 (2.3)	2 (0.8)
	Other, *n* (%)	23 (2.3)	8 (3.4)	5 (2.0)	6 (2.3)	4 (1.5)
	Unemployed, *n* (%)	660 (65.7)	109 (46.2)	184 (74.5)	148 (56.9)	219 (83.9)
Intention to work	Yes, *n* (%)	337 (33.6)	126 (53.4)	67 (27.1)	103 (39.6)	41 (15.8)
**Environmental factors**						
Neighborhood trust	Agree or somewhat agree, *n* (%)	506 (50.5)	110 (46.6)	135 (54.9)	129 (49.6)	132 (50.8)
Neighborhood norms	Agree or somewhat agree, *n* (%)	422 (42.1)	95 (40.3)	106 (43.1)	111 (42.7)	110 (42.3)
**Body Composition**						
Body fat mass, kg	Mean (SD)	15.2 (5.6)	15.3 (6.0)	14.7 (5.1)	15.6 (5.7)	15.1 (5.4)
Fat free mass, kg	Mean (SD)	41.6 (8.1)	50.3 (5.7)	46.7 (5.1)	36.5 (3.6)	34.1 (3.5)
**Physical function**						
Grip strength, kg	Mean (SD)	27.1 (7.7)	35.5 (5.6)	30.9 (5.8)	23.0 (3.7)	20.1 (3.4)
5 m usual walking speed, m/s	Mean (SD)	1.39 (0.25)	1.44 (0.23)	1.33 (0.27)	1.47 (0.24)	1.31 (0.25)
**Cognitive function**						
MoCA-J (range: 0–30)	≤25 (mild cognitive impairment), *n* (%)	650 (65.0)	150 (63.8)	195 (78.9)	116 (44.6)	189 (73.3)

GDS-5, 5-item version of the Geriatric Depression Scale; MoCA-J, the Japanese version of Montreal Cognitive Assessment; SD, standard deviation; UCLA, University of California, Los Angeles; WHO, World Health Organization.

Missing values are removed.

## STRENGTHS AND LIMITATIONS

In this study profile, we provided an overview of the baseline participant characteristics and study design of the Wako Cohort Study. This study has several strengths. First, various measurements were assessed during the baseline survey. In particular, the survey of adults aged ≥65 years included several objective measures, including body composition and physical function, in addition to subjective measures assessed in the questionnaire survey. Second, Wako City will provide data regarding several types of follow-up outcomes. Therefore, our study will provide new insights into individual and socio-environmental factors related to the extension of healthy life expectancy and the reduction of health disparities among community-dwelling adults from several perspectives.

Our study also has several limitations. First, although the response rate to the mail-in survey for those aged ≥65 years was relatively high, the number of valid respondents to the mail-in survey of participants aged 40–64 years was 2,395 (26.4% valid response rate), which is low compared to other cohort studies of middle-aged and older individuals.^30^ This implies that the age group 40–64 years might be more biased in favor of healthier people, which may introduce bias in the findings and may affect the comparisons of indicators between the age groups. Therefore, we cannot completely rule out the possibility of a selection bias. Second, participants in the on-site survey were not selected randomly from the study population, and they were relatively healthy older persons who were able to participate in the assessments at the community center on their own and might have had higher health consciousness. In fact, the medical and lifestyle characteristics in the on-site survey were more favorable than those in the mail-in survey. In addition, the HIS scores for the on-site survey tended to be higher than those for the mail-in survey, as shown in Table 2 and Table 3. Thus, the potential for the healthy volunteer effect needs to be taken into account.

In conclusion, the Wako Cohort Study was launched in 2023 to identify individual and socio-environmental factors related to the extension of healthy life expectancy and the reduction of health disparities among community-dwelling adults. The Wako Cohort Study consists of two surveys: a mail-in survey for those aged ≥40 years and an on-site survey for those aged ≥65 years. We plan to conduct a follow-up survey and collect data on several outcomes, including mortality, LTCI certification, and long-term care costs. The Wako Cohort Study is expected to provide new insights into establishing effective population-based health promotion and preventive long-term care strategies that can be applied to diverse populations.
